# Explanted Diseased Livers – A Possible Source of Metabolic Competent Primary Human Hepatocytes

**DOI:** 10.1371/journal.pone.0101386

**Published:** 2014-07-07

**Authors:** Moritz Kleine, Marc Riemer, Till Krech, Daphne DeTemple, Mark D. Jäger, Frank Lehner, Michael P. Manns, Jürgen Klempnauer, Jürgen Borlak, Hueseyin Bektas, Florian W. R. Vondran

**Affiliations:** 1 ReMediES, Department of General, Visceral and Transplantation Surgery, Hannover Medical School, Hannover, Germany; 2 Institute of Pathology, Hannover Medical School, Hannover, Germany; 3 Department of Gastroenterology, Hepatology and Endocrinology, Hannover Medical School, Hannover, Germany; 4 German Centre for Infection Research (DZIF), partner site Hannover-Braunschweig, Hannover, Germany; 5 Center of Pharmacology and Toxicology, Hannover Medical School, Hannover, Germany; St. Luc University Hospital, Belgium

## Abstract

Being an integral part of basic, translational and clinical research, the demand for primary human hepatocytes (PHH) is continuously growing while the availability of tissue resection material for the isolation of metabolically competent PHH remains limited. To overcome current shortcomings, this study evaluated the use of explanted diseased organs from liver transplantation patients as a potential source of PHH. Therefore, PHH were isolated from resected surgical specimens (Rx-group; n = 60) and explanted diseased livers obtained from graft recipients with low labMELD-score (Ex-group; n = 5). Using established protocols PHH were subsequently cultured for a period of 7 days. The viability and metabolic competence of cultured PHH was assessed by the following parameters: morphology and cell count (CyQuant assay), albumin synthesis, urea production, AST-leakage, and phase I and II metabolism. Both groups were compared in terms of cell yield and metabolic function, and results were correlated with clinical parameters of tissue donors. Notably, cellular yields and viabilities were comparable between the Rx- and Ex-group and were 5.3±0.5 and 2.9±0.7×10^6^ cells/g liver tissue with 84.3±1.3 and 76.0±8.6% viability, respectively. Moreover, PHH isolated from the Rx- or Ex-group did not differ in regards to loss of cell number in culture, albumin synthesis, urea production, AST-leakage, and phase I and II metabolism (measured by the 7-ethoxycoumarin-*O*-deethylase and uracil-5′-diphosphate-glucuronyltransferase activity). Likewise, basal transcript expressions of the CYP monooxygenases 1A1, 2C8 and 3A4 were comparable as was their induction when treated with a cocktail that consisted of 3-methylcholantren, rifampicin and phenobarbital, with increased expression of CYP 1A1 and 3A4 mRNA while transcript expression of CYP 2C8 was only marginally changed. In conclusion, the use of explanted diseased livers obtained from recipients with low labMELD-score might represent a valuable source of metabolically competent PHH which are comparable in viability and function to cells obtained from specimens following partial liver resection.

## Introduction

Despite considerable improvements achieved in the cryopreservation and hypothermic storage of primary human hepatocytes (PHH) [1]–[4] the availability of PHH for cell biology use and hepatocyte transplantation (HCTx) remains insufficient due to the limited number of adequate donors.

Specifically, the demand for PHH has continuously increased due to tightened standards in drug testing [5], [6] and an expanded use of primary cells in basic and clinical research [7], [8]. On the other hand, large specimens from liver resections are less frequently available since extended liver surgery is less readily performed as a result of advancement in surgical techniques. Of note, while the widespread use of laparoscopic liver surgery is considered to be less harmful to the patient, the longer overall resection times result in prolonged warm ischemia time of resected liver tissue [9], [10]. Furthermore, rejected donor organs now take a back seat as potential sources for PHH since extended criteria for the acceptance of donor livers for solid organ transplantation are applied routinely [11]. Consequently, the existing gap between supply and demand of PHH will widen in the near future.

We and others demonstrated repeatedly the utility of liver specimens obtained during partial liver resection (performed for varying reasons) as a valuable source of PHH [12]. Even marked histopathological alterations of the liver tissue such as cholestasis, steatosis or fibrosis still allow for the isolation of metabolically competent PHH although cell yields might be significantly reduced [10]. Furthermore, the clinical condition of the tissue donors, as indicated by liver-specific markers, does affect the metabolic competence of isolated PHH to a certain extent [12], [13]. Nonetheless, PHH of slightly ‘sub-optimal’ quality might still be useful in biomedical research.

Based on these considerations, the use of explanted diseased organs as a source of liver tissue for the isolation of PHH may overcome current limitations. However, only very limited data is available on the quality of PHH isolated from such tissue sources. Recently, it has been shown that the isolation of PHH from explanted livers seems feasible [14], and that the isolated cells might be usable for HCTx [15]. Kehr et al. have demonstrated that modifications of the isolation techniques might even enable large scale liver cell isolation from entire explanted organs [16].

Therefore, the aim of the present study was to further evaluate the potential of explanted diseased livers for the isolation of primary human hepatocytes. Due to the broad spectrum of indications for LTx our focus was placed on a subgroup of graft recipients with low labMELD-scores (e.g. due to approved exceptions for transplantation) as tissue donors. Here we show that the use of these so far disregarded tissues as a source of PHH may overcome current shortages of supply with cell viabilities and metabolic competences of hepatocytes being comparable to those obtained from specimens following partial liver resection.

## Methods

### Liver specimen & clinical parameters

Liver tissue was obtained after partial hepatectomy (Rx-group; excluding laparoscopic liver resections) or from explanted whole livers following LTx (Ex-group) and directly transferred to the laboratory for immediate cell isolation (time delay between hepatectomy and perfusion <1 h). All tissue donors gave written informed consent for experimental use of clinical data and liver specimen prior to surgery. The protocol was approved by the ethics commission of Hanover Medical School. The following data from routinely performed preoperative blood tests were analyzed: alkaline phosphatase (AP), alanine-aminotransferase (ALT), amino-aspartatetransaminase (AST), total bilirubin (t-bil), cholinesterase (CHE), γ-glutamyltransferase (γ-GT), serum creatinine, international normalized ratio (INR) and quick value. The labMELD-score was calculated according to the following formula: (0.957xLN(creatinine) +0.378xLN(t-bil) +1.12xLN(INR) +0.643)x10 [17].

### Hepatocyte isolation and culture

Hepatocyte isolation was performed using a modified 2-step collagenase perfusion technique as previously reported [12]. Briefly, the liver specimen was cannulated under sterile conditions and flushed once with 500 ml washing buffer containing 2.5 mM EGTA (SIGMA-Aldrich). This was followed by perfusion with 100 ml digestion buffer containing 0.05% collagenase P (Roche Diagnostics) allowing recirculation of the perfusate. The resulting cell suspension was poured through a gauze-lined funnel and centrifuged with subsequent washing of the cell pellet using ice-cold PBS (50 g, 5 min, 4°C). Cells were then re-suspended in William's medium E (Biochrom AG) supplemented as previously reported [2]: 1 µM insulin, 1 µM dexamethason/fortecortin, 100 U/ml penicillin, 100 µg/ml streptomycin, 1 mM sodium pyruvate, 15 mM HEPES buffer, 4 mM L-glutamine and 5% FCS. Cell number and viability were determined by the Trypan blue exclusion test. Hepatocytes were cultured using 6-well plates precoated with a single layer of rat tail collagen. The latter was extracted as previously reported [18]. Cells were seeded at a concentration of 2×10^6^ viable cells per well. Sixteen to eighteen hours after plating, culture medium was changed to remove dead and non-adherent cells. Hepatocytes were cultured for a period of 7 days with daily change of culture medium. Culture supernatants and cell pellets were collected on days 1, 3, 5 and 7 and stored at −80°C until analysis in batch.

### Albumin synthesis

The synthesis of albumin by PHH was assessed using the Human Albumin ELISA Quantitation Set (Bethyl Laboratories) according to the manufacturer's instructions.

### Aspartate-aminotransferase activity and urea production

The activity of the aspartate-aminotransferase (AST) served as a measure for the degree of cell damage while the production of urea served as an indicator for ammonia detoxification. Both parameters were determined in the supernatants of hepatocyte cultures by standardized procedures (Roche Molecular Diagnostics) performed by the central laboratory of Hanover Medical School.

### Gene expression

RNA was isolated using the NucleoSpin RNA2-Kit (Machery-Nagel) according to manufacturer's recommendations. 1 µg of total RNA was used for reverse transcription with the Omniscript kit (Qiagen). Real-Time PCR was performed with 200 ng of cDNA product on a StepOne Plus real-time PCR system (Applied Biosystems). To determine relative expression levels of target genes, Ct values were normalized against house-keeping gene β-actin using the δCt value to calculate relative expression [19]. The following primers/probes (Applied Biosystems) were used: β-actin: Hs99999903_m1; albumin: Hs00910225_m1. Cytochrome P450 (CYP) 1A1: Hs01054797_g1, 2C8: Hs02383390_s1, 3A4: Hs00604506_m1.

Transcript expression of albumin and basal expression of CYPs 1A1, 2C8 and 3A4 in cultured human hepatocytes were monitored throughout the entire culture period (days 1, 3, 5 and 7). Furthermore, the inducibility of CYP isoforms was studied on day 7 in culture following repeated treatment with a cocktail that consisted of 2.5 µM 3-methylcholantren, 5 µM rifampicin and 2 mM phenobarbital for 72 h. CYP mRNA-expression after induction of ECOD was determined as x-fold change compared to unstimulated control cultures.

### Phase I & II metabolism

Phase I metabolism was investigated by quantification of the 7-ethoxycoumarin-*O*-deethylase (ECOD) as previously reported [12]. In brief, induction of ECOD activity was studied applying the drug cocktail (3-MC, rifampicin, phenobarbital) as described above followed by exposure of PHH to 25 µM ethoxycoumarin in combination with 1.5 mM salicylamide and 2 mM probenecid for 2 h. The fluorescent product 7-hydroxycoumarin was then determined from culture supernatants as originally described by Greenlee and Poland [20] using an excitation wavelength of 386 and an emission wave length of 460 nm, respectively. For differentiation of total and free 7-hydroxycoumarin ( =  not glucuronidated), aliquots of supernatants were processed with and without pre-treatment with 100 IU of β-glucuronidase for 1 h at 37°C (all SIGMA-Aldrich). Phase II enzyme reactions likewise were investigated on day 7 of culture by uracil-5′-diphosphate-glucuronyltransferase (UDP-GT) activity using 4-methylumbelliferone (4-MU) as a substrate as previously reported [12].

### *In vitro* quantification of cell numbers

For quantification of cell numbers cultured PHH were detached from 6-well plates by applying collagenase P solution (0.05%) for 30 min and cells were spun down at 200 g. Following re-suspension in 2 ml PBS, 20 µl of this suspension were again centrifuged and the resulting cell pellet then stored at −80°C to await DNA analysis in batches. A standard curve for DNA quantification was generated from freshly isolated PHH and DNA quantification was related to the total of cells cultured in a well using the CyQuant Cell Proliferation Assay Kit (Invitrogen) according to the manufacturer's instructions.

### *In vitro* morphology & Histopathology

The morphology of the cells attached to the collagen-coated plates was assessed daily using conventional phase-contrast microscopy. Histopathological assessment was done with HE-stained slides of FFPE-tissue-specimens. Special stains (e. g. PAS, Elastica van Gieson, Gomori, Pearls, Rhodanin) were performed on representative sections or whenever necessary.

### Statistical analysis

Statistical analysis was performed using IBM SPSS Statistics 21.0. The Mann-Whitney-U test, Kruskal-Wallis test and one-way ANOVA were applied as appropriate. Differences were regarded statistically significant with p<0.05. Results were expressed as mean ±SEM unless otherwise indicated.

## Results

### General donor characteristics

**Figure 1:**
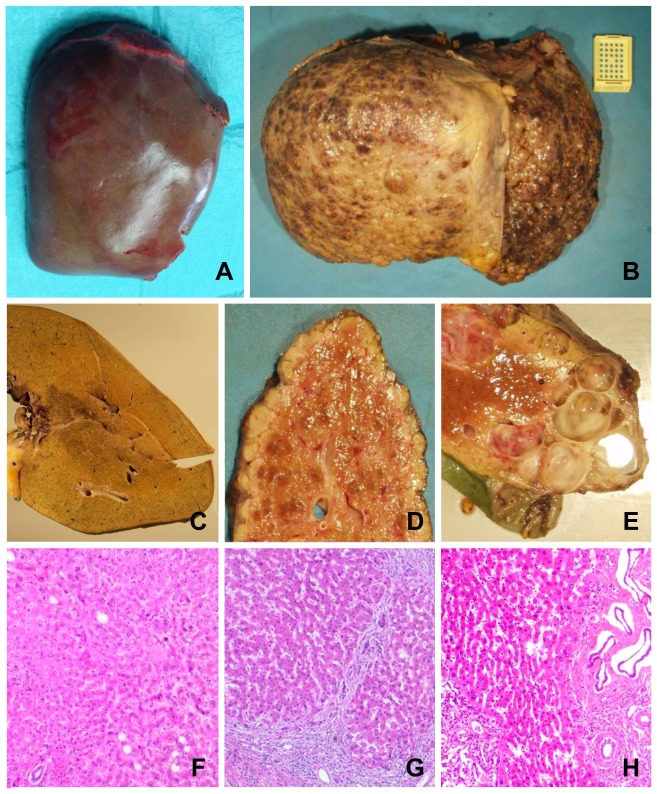
Macroscopic and microscopic appearance of liver tissue used for hepatocyte isolation from resected surgical specimen and explanted diseased livers. Representative macroscopic appearances of liver preparations removed during partial hepatectomy (lobectomy right) due to Klatskin-Tumor (A) and a liver explant due to hepatocellular carcinoma (HCC) (B) already showing marked differences in terms of cirrhotic alterations in the latter. The corresponding cut surfaces and representative histologic sections show homogenous liver parenchyma without any significant pathological finding in the former (C, F) and a mixed micro- and macronodular cirrhosis with mild chronic inflammation in the latter (D, G). Several smooth walled cysts are apparent on the cut surface of an explant of a polycystic liver (E). A corresponding histological section shows areas of fibrosis with cystically dilated bile duct structures with intervening areas of inconspicuous liver parenchyma (H).

Due to the severity of the underlying diseases that eventually necessitated LTx, the macroscopic appearance of the explanted organs was grossly altered as compared to the liver specimens obtained from partial hepatectomy (*Figures 1A–E*). The histopathology of explanted livers revealed clear signs of fibrosis, cirrhosis or cystic alteration (*Figures 1F–H* *)* while specimens obtained from liver resections due to benign diseases or secondary liver tumors contained normal liver parenchyma. Note, even after chemotherapy significant alterations in non-tumorous tissue was rarely observed possibly as a result of the recovery period of a minimum 4 weeks between the last chemotherapy cycle and the performed surgery. Hepatic steatosis at varying degrees was observed among all entities leading to either liver resection or transplantation (data not shown). Nonetheless, no statistically significant differences in liver function tests were observed between the Rx- and Ex-group and likewise the labMELD-score did not differ (*Table 1*). However, stratification of tissue donors by indication for surgery rather than type of surgery revealed significant (p<0.05) higher activities of serum AP, ALT, AST, γ-GT and t-bil for primary liver tumors compared to all other underlying diseases (data not shown). Note, tissue donors of the Ex-group were significantly younger but well within the range of the Rx-group (*Table 1*). Age was therefore not a confounder in the isolation outcome upon further analyses.

**Table 1:** Demographics and clinical data of tissue donors for cell isolation.

	Resection group (Rx)	Explant group (Ex)
**Total number of patients**, n	60	5
**Gender**, n [f/m]	21/39	2/3
**Age at time of surgery**, years (range)	59.2±2.5 (19–79)	45.6±4.6 (30–54) *
**Indication for surgery**, n (% of total)		
Primary liver tumor [HCC/CCC]	8/14 (37)	2/0 (40)
Secondary liver tumor [CRC/miscellaneous]	20/11 (52)	0/0 (0)
Benign liver lesion	6 (10)	0 (0)
Primary sclerosing cholangitis	0 (0)	1 (20)
Polycystic liver disease	1 (2)	2 (40)
**Lab values at time of surgery**		
AST, U/l	49.4±7.8	61.8±17.3
ALT, U/l	44.7±7.3	54.4±15.3
AP, U/l	145.0±23.3	203.6±52.3
γ-GT, U/l	191.4±34.4	203.2±63.8
Total bilirubin, µmol/l	18.8±5.6	25.2±12.3
CHE, kU/l	7.5±0.3	5.2±1.3
Creatinine, µmol/l	74.6±3.5	83.4±16.3
INR, (ratio)	1.1±0.02	1.1±0.03
Quick value, %	89.5±1.8	82.6±4.1
**Lab-MELD at time of surgery** (range)	8.3±0.4 (5.4–18.4)	10.1±1.3 (6.9–13.2)

f =  female, m =  male; HCC  =  hepatocellular carcinoma; CCC  =  cholangiocellular carcinoma; CRC  =  colorectal cancer; AST  =  aspartate-aminotransferase; ALT  =  alanine-aminotransferase; AP  =  alkaline phosphatase; γ-GT  =  Gamma-glutamyltransferase; CHE  =  cholinesterase; INR  =  international normalized ratio; Data presented as MEAN ±SEM unless otherwise indicated;

* = p<0.05.

### Cell yield & viability

An isolation of hepatocytes was successfully performed with all surgical specimens (*n* = 65). Following a collagenase perfusion time of 18.4±0.5 min a mean yield of 160.0±15.2×10^6^ hepatocytes with an average viability of 83.7±1.5% could be obtained. This corresponds to a final isolation outcome of 5.0±0.5×10^6^ viable hepatocytes/g liver. By trend, more viable cells per isolation were obtained in the Ex-group with overall comparable cell viability. Since significantly larger tissue samples (2-fold; p = 0.001) were used for cell isolation in the latter, final yields of viable hepatocytes/g liver in this group though eventually were lower than in the Rx-group but this was not statistically significant (*Table 2*).

**Table 2:** Results of cell isolation.

	Resection group (Rx)	Explant group (Ex)	p-value
**Number of cases**, n	60	5	-
**Weight of liver sample**, g	31.8±2.6	61.6±6.1	*0.001*
**Time of collagenase perfusion**, min	18.7±0.5	16.6±1.3	0.128
**Number of total cells**, × 10^6^	190.8±20.0	231.3±45.5	0.294
**Number of viable cells**, × 10^6^	158.7±16.2	176.7±42.2	0.479
**Cell viability**, %	84.3±1.4	76.0±8.6	0.251
**Total cells/g liver**, × 10^6^	6.0±0.8	3.7±0.6	0.337
**Viable cells/g liver**, × 10^6^	5.3±0.7	2.9±0.7	0.212

Data presented as MEAN ±SEM unless otherwise indicated.

Noteworthy, despite differences in liver function tests among individual tissue donors, no relevant influence on the isolation outcome was observed. Specifically, for the aforementioned subgroup of primary liver tumors on average 4.7±1.1×10^6^ viable hepatocytes/g liver and a viability of 81.0±3.1% were obtained.

### Cell morphology of cultured hepatocytes

**Figure 2:**
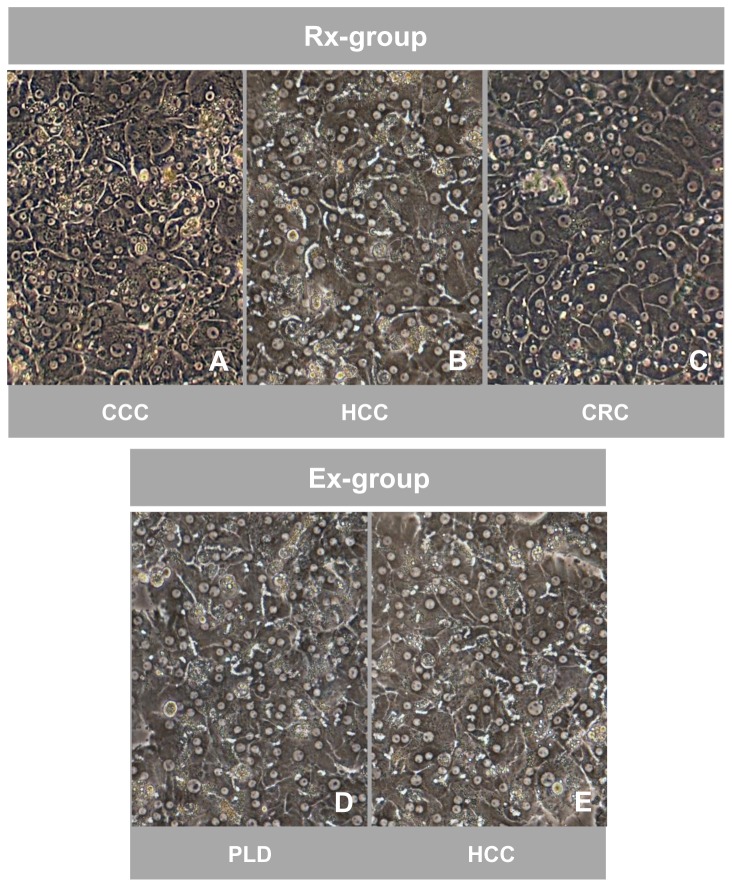
Comparable morphological appearance of cultured hepatocytes isolated from resected surgical specimens and explanted diseased livers. Phase-contrast microscopy of cultured (day 5) primary human hepatocytes isolated from resected surgical specimens (Rx-group) removed due to metastasis of colorectal cancer (CRC) (A), hepatocellular carcinoma (HCC) (B) and cholangiocellular carcinoma (CCC) (C) as well as from explanted diseased livers (Ex-group) due to HCC (D) and polycystic liver disease (PLD) (E). Hepatocytes of both groups show the typical polygonal shape, are highly prismatic and either mono- or polynucleated. Signs of the formation of bile canaliculi are present. Magnification 100x.

Detailed analysis of PHH in culture was performed with *n* = 5 specimens from the resection- (Rx) and explant-group (Ex), respectively. The cells attached to the collagen-coated plates showed the typical morphological appearance of primary human hepatocytes using phase-contrast microscopy. These were highly prismatic, presented a typical polygonal shape and were either mono- or polynucleated. Furthermore, formation of bile canaliculi was visible. No obvious differences in morphology between cultured PHH isolated from the Rx- or Ex-group were observed (*Figure 2*).

### Hepatocellular damage, albumin synthesis, urea production & cell number

**Figure 3:**
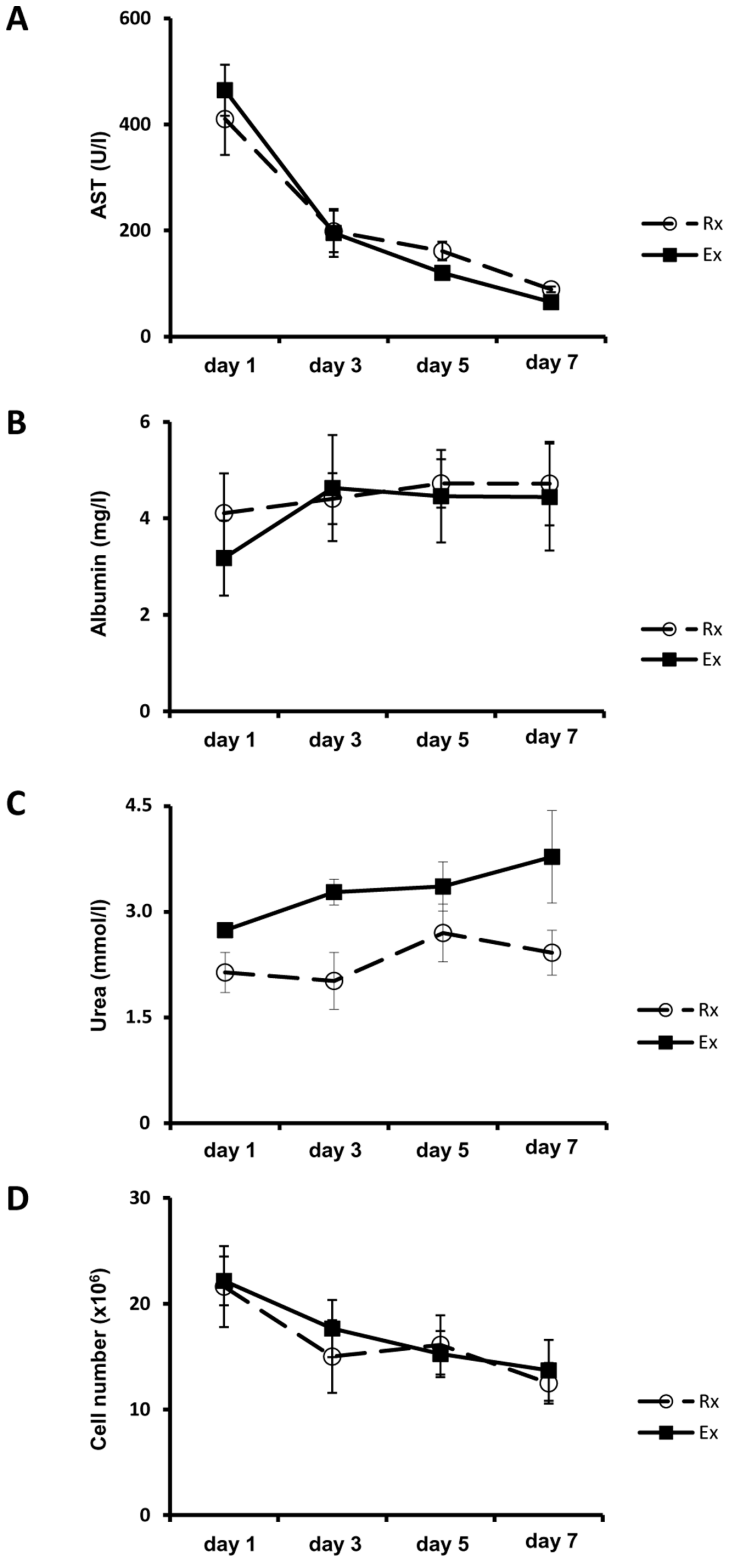
Comparable courses of AST-leakage, albumin synthesis, urea production and *in vitro* cell number in hepatocytes isolated from resected surgical specimens and explanted diseased livers. Diagrams depicting the courses of AST-leakage (A), albumin synthesis (B) and urea production (C) determined from culture supernatants on days 1, 3, 5 and 7 (following daily change of the culture medium) for the resection-group (Rx, white circles) and explant-group (Ex, black squares), respectively. Subsequent analysis of the *in vitro* cell numbers (D) by CyQuant assay was performed using the appropriate cell pellets. Data is presented as mean ±SEM of n = 5 experiments.

As a measure of cell damage, the leakage of AST into the culture supernatants was assayed but did not reveal marked differences between the Rx- and Ex-group: Following a peak in activity on day 1 of culture a continuous decline of AST-activities was observed. PHH obtained from explanted organs tended to display higher peaks on day 1 but this was not statistically significant (*Figure 3A*).

Regarding the synthesis of albumin, both groups reached similar levels of albumin production on days 3 to 7. For hepatocytes from resected liver specimens albumin synthesis was almost constant throughout the entire culture period whereas for liver cells of the Ex-group production levels were initially lower (*Figure 3B*). However, the difference was not of statistical significance.

Production of urea as an indicator for ammonia detoxification was likewise determined. Cells of both groups produced increasing urea levels in the course of culture time thus indicating establishment of stable cell function of cultured PHH (*Figure 3C*). The constantly higher production of urea observed with the Ex-group was not statistically significant.

Moreover, the number of PHH was monitored throughout the entire culture period using the CyQuant assay. This assay is based on the fluorometric detection of DNA and revealed comparable results for both groups of cultured hepatocytes with the number of cells slowly and continuously decreasing over time (*Figure 3D*).

### Transcript expression of albumin and CYP genes

**Figure 4:**
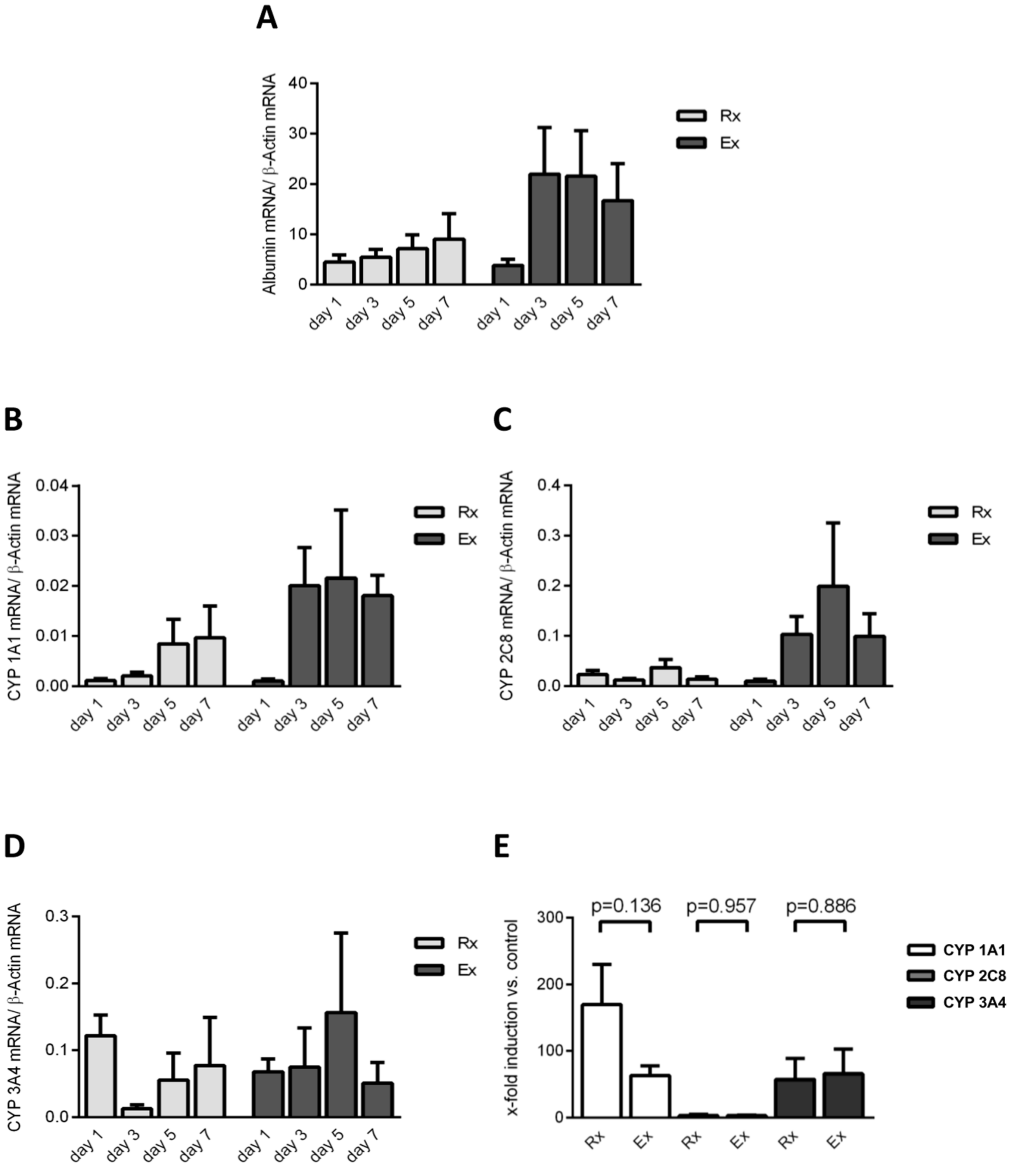
Transcript expression of albumin and CYP genes are comparable in hepatocytes isolated from resected surgical specimens and explanted diseased livers. Diagram depicting the mRNA-expression of albumin (A) determined by RT-PCR on days 1, 3, 5 and 7 (Rx: light grey bars, Ex: dark grey bars). Furthermore, the courses of basal expression of cytochromes P450 (CYP) 1A1 (B), 2C8 (C) and 3A4 (D) are shown for the resection-group (Rx, light grey bars) and explant-group (Ex, dark grey bars), respectively. Following induction of the 7-ethoxycoumarin-*O*-deethylase (ECOD) activity by exposing the cells to 3-methylcholantren, rifampicin and phenobarbital for 72 h, courses of mRNA-expression in relation to the unstimulated control are shown in both groups for CYPs 1A1 (white bars), 2C8 (light grey bars) and 3A4 (dark grey bars) (E). Data is presented as mean ±SEM of n = 5 experiments.

Real-time RT-PCR evidenced an approximately 4-fold increase of albumin transcript expression between days 1 and 3 for the Ex-group and was constantly higher as compared to the Rx-group. The latter likewise showed increasing expression levels during culture but to a lesser magnitude (*Figure 4A*). There was agreement between albumin gene expression and its production as determined in culture supernatants using an established ELISA quantification method.

Basal and induced expression of CYPs 1A1, 2C8 and 3A4 mRNA were determined on days 1, 3, 5 and 7 of culture. For CYPs 1A1 and 2C8 apart from day 1 higher mRNA-expression levels were observed with the Ex-group as compared to the Rx-group, nonetheless did not reach statistical significance (*Figures 4B* *+C*). Real-Time RT-PCR further revealed stable expression of CYP 3A4 throughout the entire study period with again no significant differences among the two groups (*Figure 4D*). Importantly, treatment of PHH cultures with a drug cocktail consisting of 3-methylcholantren, rifampicin and phenobarbital induced mRNA-expression of CYPs 1A1 and 3A4 significantly (at least 57-fold) (*Figure 4E*). The extent of CYP 2C8 induction was much less when compared to the other isoforms studied (3.0±1.8 and 3.1±0.7-fold increases for Rx- and Ex-group, respectively) and the expression levels of CYPs 2C8 and 3A4 mRNA were comparable between both groups (*Figure 4E*). Overall, drug treatment caused strong induction in CYP 1A1 mRNA-expression but did not reach statistical significance due to the well-known high inter-individual differences [21] among donors.

### Metabolic assays

**Figure 5:**
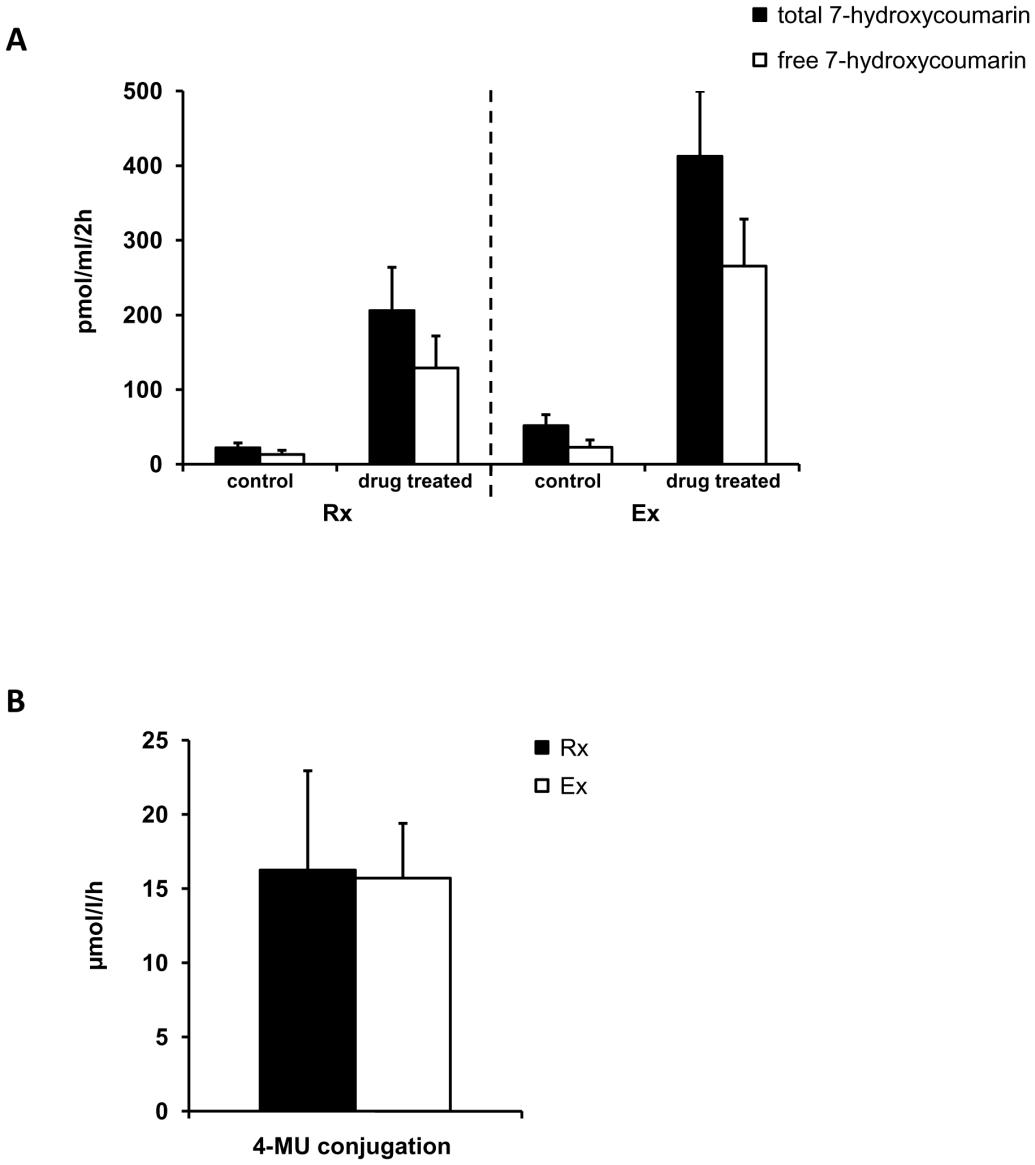
Phase I and II enzyme reactions are comparable in hepatocytes isolated from resected surgical specimens and explanted diseased livers. Diagrams depicting the activity of the 7-ethoxycoumarin-*O*-deethylase (ECOD) (A) in PHH of the resection-group (Rx) and explant-group (Ex) regarding the total 7-hydroxycoumarin as a product (black bars) as well as the non-glucuronidated form (free 7-hydroxycoumarin; white bars). Untreated controls were compared to PHH exposed to 3-methylcholantren, rifampicin and phenobarbital for 72 h ( =  drug treated). Furthermore, the uracil-5′-diphosphate-glucuronyltransferase (UDP-GT) activity (B) using 4-methylumbelliferone (4-MU) as a substrate is shown for the Rx- (black bar) and Ex-group (white bar), respectively. Data is presented as mean ±SEM of n = 5 experiments.

Drug induced ECOD activity as a measure for phase I enzyme reactions was comparable among both groups as seen by approximately 8-fold and 9-fold induction for the Rx- and Ex-groups, respectively (*Figure 5A*). Furthermore, treatment of the appropriate supernatants with β-glucuronidase revealed significant glucuronidation of the metabolic product 7-hydroxycoumarin (*Figure 5A*). However, the glucuronidation activities did not differ significantly between the Rx- and Ex-group. We further investigated Phase II enzyme activities (UDP-GT) by assaying 4-MU glucuronidation. Again, PHH of the Rx- and Ex-group displayed comparable enzyme activities, i.e. 16.3±6.7 vs. 15.7±3.7 µmol/l/h, respectively (*Figure 5B*).

## Discussion

Over the years the demand for primary human hepatocytes for biomedical research and the development of cellular based therapies has risen continuously but cannot be met by the tissue available for PHH isolation. Additionally, changes in surgical procedures, i.e. less frequently performed large liver tissue resections, and criteria for rejection of donor organs for transplantation further limit tissue supply. We therefore explored the use of explanted diseased organs available from LTx patients as an alternative source for metabolically competent PHH.

Indeed, overcoming shortages in supply of human liver tissue donations is a major challenge to biomedical researchers and necessitated new approaches for the development of alternative testing strategies. Apart from the considerable progress made in the cryopreservation or hypothermic storage of PHH [1]–[3], [22] stem cell derived hepatocytes are the focus of many research groups. However, the cryopreservation of hepatocytes is associated with a substantial loss of viable cells during thawing and a decline in metabolic competences as compared to freshly isolated cells [23]. Thus, while cryopreservation improves logistical problems and allows prolonged storage of PHH this will not decisively increase the quantity of overall available high quality liver cells.

Extensive research efforts have therefore been invested to generate mature hepatocytes from immortalized cell lines as well as hepatic progenitor cells. Promising results have been achieved with the cell line cBAL111, as these cells can produce urea, albumin and cytokeratin 18, and eliminate galactose [24]. However, cell line-derived hepatocytes display considerable heterogeneity in their functional capabilities compared to PHH and thus continue to play a minor role. Furthermore, progenitor cells that are quiescent in the healthy liver and become activated in certain liver diseases in which the regenerative capacity of mature hepatocytes is impaired might represent a promising option for *in vitro* hepatocyte generation. Unfortunately, although reports describing such cells are numerous there is no consensus regarding their phenotypical cellular identity, and thus a broader usage is still lacking [25].

Hence, conventional hepatocyte isolation from human liver tissue remains the most reliable and best accessible source of well characterized and highly functional PHH to date. Any approach increasing the availability of liver tissue suitable for cell isolation would thus directly improve the supply with PHH – at least until protocol optimization of the above mentioned procedures have been achieved and become routinely available.

We therefore explored the utility of PHH isolated from explanted diseased organs. As expected, tissue samples obtained after LTx displayed marked signs of histopathological alteration (*Figure 1*) whereas tissue quality of livers undergoing partial liver resection was free of gross morphological alterations. This is particularly true in cases where secondary tumors (leading diagnosis in this study) and benign liver diseases were indications for surgery. Nevertheless, we and others have previously reported [10], [12] that the presence of such alterations does not necessarily result in functional impairment of PHH but rather affects the isolation outcome in terms reduced cell yields. Due to the fact that hepatocyte viabilities as well as cell numbers in culture and markers of injury (AST) were comparable for the Rx- and Ex-groups a comparison of the metabolic competence amongst the different sources of PHH thus was attempted. Using a diverse set of data (gene expression of CYPs, albumin synthesis, various metabolic assays) comparable metabolic competences of cultured hepatocytes were determined. Notably, our data confirms the only previously published report on the use of diseased liver tissue for hepatocyte isolation [14]. In their study, the authors reported diverse indications for surgery, especially due to underlying biliary cirrhosis or alcoholic liver disease that would still allow for an isolation of PHH. This initial investigation therefore stimulated the use of diseased liver tissue as valuable source of PHH. Nonetheless, the study suffered from some methodological weaknesses. First of all, the protocol applied resulted in a very low mean isolation outcome of only 0.35×10^6^ total cells with a viability of about 40% as compared to 194×10^6^ total cells and 84% viability obtained in the present study. Secondly, the authors measured albumin synthesis and urea production as the only functional parameters while phase I and II metabolism as a major constituent of liver function was not investigated at all. Finally, the study of Bhoghal et al. [14] lacked detailed clinical information that would be required to classify tissue donors by severity of the underlying disease and is especially important for the correct interpretation of experimental data regarding the transplant recipients (e.g. labMELD-score). Noteworthy, a further study on explanted livers as a source of PHH was recently published by Gramignoli et al. [15]. As their data mainly resulted from LTx due to metabolic diseases and primarily was confined to children, their findings consequently are not directly comparable with the study results of the present investigation. Nonetheless, these livers were still considered to be useful sources for the isolation of metabolically competent PHH, at least in part. These data highlight the potential use of so far disregarded tissues as a source for human hepatocytes.

However, the following caveats need to be considered. Obviously, the severity of the underlying disease is a central issue. While histomorphological changes such as cirrhosis or steatosis are known to impair the cell yield of viable PHH, increasing loss of liver function as observed in patients eventually receiving a transplant likewise might influence isolation outcome or function of PHH. We therefore focused on the use of liver tissue from graft recipients that were transplanted with a rather low labMELD-score of 10.1 on average (and thus within the range of the tissue donors undergoing partial hepatectomy), mainly due to approved exceptions for LTx (3 out of 5 patients) [26]. Given the fact that at least in the Eurotransplant area a significant increase in mean MELD-score at the time of organ allocation is common [27], it remains unclear whether such severely harmed livers are suitable for isolation of metabolically competent hepatocytes. If the metabolic competence was not affected in PHH isolated from these organs but rather the number of viable cells obtained, choosing a different technical approach than currently applied for procurement of resected specimens might enable adequate cell yields from these tissues (e.g. using the whole organ perfusion technique described by Kehr et al. [16]). Consideration should also be given to the fact that the cohort of critically-ill patients regularly receives multiple drug treatment prior to LTx that might impose limitation for their subsequent use in toxicological studies. Finally, the use of explanted organs is associated with considerable logistical efforts: Unlike partial liver resections that are regularly planned for the day-to-day routine, liver transplantations will be performed immediately whenever suitable organs are available, and this often occurs outside core surgery times.

On the other hand, the use of explanted diseased organs as a source of PHH might offer new possibilities for basic research and subsequent development of therapeutic strategies. PHH from tissue donors suffering from hepatitis B or C could be used to establish novel *in vitro* hepatitis models, especially concerning the highly resistant genotypes [28]. Liver cells isolated from organs of severely ill patients (high labMELD-score) could undergo detailed phenotypical analysis for identification of activated progenitors cells in order to further study the regenerative capacity of the liver under these conditions [25]. Furthermore, autologous liver cell preparations from explanted organs could be used to develop innovative immunological treatment concepts in the setting of solid organ transplantation such as liver re-population [29].

In conclusion, explanted diseased livers represent a valuable source of metabolically competent primary human hepatocytes. Considering appropriate patient selection (low labMELD-score, approved exceptions), a PHH quality comparable to cells obtained from specimens following partial liver resection can be regularly obtained while overall cell yields might be slightly lower. Studies with a focus on patients undergoing liver transplantation with high labMELD-score are now required to further extend the pool of potential tissue donations for liver cell isolation.
